# 10 The Impact of Insurance Disparities on Long-term Burn Outcomes: A Burn Model System Investigation

**DOI:** 10.1093/jbcr/irac012.014

**Published:** 2022-03-23

**Authors:** Manuel Castillo-Angeles, Lauren J Shepler, Gretchen J Carrougher, Nicole S Gibran, Barclay T Stewart, Steven E Wolf, Karen J Kowalske, Colleen M Ryan, Jeffrey C Schneider, Anupama Mehta

**Affiliations:** Brigham and Women's Hospital, Boston, Massachusetts; Spaulding Rehabilitation Hospital, Boston, Massachusetts; Department of Surgery, The University of Washington, Seattle, Washington; University of Washington, Wellfleet, Massachusetts; University of Washington, Seattle, Washington; University of Texas Medical Branch at Galveston, Galveston, Texas; Department of Surgery, The University of Texas Southwestern Medical Center, Dallas- Fort worth, Texas; Harvard Medical School, Boston, Massachusetts; , Massachusetts; Brigham and Women's Hospital, Quincy, Massachusetts

## Abstract

**Introduction:**

Access to healthcare and insurance coverage are associated with quality of life, morbidity, and mortality outcomes. However, most studies have only focused on same-admission and short-term outcomes due to the lack of national longitudinal data and there is limited data on this topic in the burn literature. Our aim was to determine the effect of insurance status on long-term outcomes in a national sample of burn patients.

**Methods:**

This is a retrospective study using the longitudinal Burn Model System National Database from January 2015 to April 2021. The inclusion criteria were all adult patients admitted for burn injury from participating sites. Main outcomes were the physical (PCS) and mental (MCS) health component summary scores of the Veterans RAND 12 (VR-12) score at 6, 12, and 24 months after injury. Multivariable regression was used to examine the association between insurance status and the outcomes, adjusting for demographics (i.e., age, gender, race/ethnicity) and burn injury severity.

**Results:**

A total of 3,698 burn patients were included. Mean age was 43.39 (SD 15.84) years, 72% were male and 76% were white. Most patients had private/commercial insurance (56.37%), followed by Medicare (14.42%) and Medicaid (13.18%). The remaining 16% were uninsured patients (self-pay or philanthropy). Mean PCS scores were 43.64 (SD 10.87), 45.31 (SD 11.04) and 46.45 (SD 10.65) and Mean MCS scores were 47.80 (SD 12.35), 48.18 (SD 12.30) and 48.44 (SD 12.18) at 6, 12 and 24 months, respectively. In adjusted analyses, Medicaid insurance was associated with worse MCS at 6 months (Coefficient -3.90, p=0.001), and worse PCS at 12 and 24 months (Coefficient -3.09, p=0.004 and Coefficient -4.18, p< 0.001, respectively), compared to uninsured status. Medicare insurance was associated with worse PCS scores at 24 months (Coefficient -3.07, p=0.013).

**Conclusions:**

Having Medicaid and Medicare insurance was significantly associated with a lower health-related quality of life at long-term follow up, even after adjusting for demographics and burn injury severity. Further studies need to focus on analyzing the reasons for these disparities and developing strategies to improve the quality of life of this subpopulation.

